# Evaluation of VEGF, FGF and PDGF and Serum Levels of Inflammatory Cytokines in Patients with Glioma and Meningioma in Southern Iran

**DOI:** 10.31557/APJCP.2019.20.10.2883

**Published:** 2019

**Authors:** Seyedeh Azra Shamsdin, Ali Mehrafshan, Seyed Mohammad Rakei, Davood Mehrabani

**Affiliations:** 1 *Gastroenterohepatology Research Center,*; 3 *Department of Neurosurgery, Shiraz University of Medical Sciences, Shiraz,*; 2 *Department of Neurosurgery, Qom University of Medical Sciences, Qom, Iran. *

**Keywords:** Angiogenic factors, inflammatory cytokine, meningioma, Glioma

## Abstract

**Background:**

Meningioma and glioma are common central nervous system tumors. Hypoxic tumor cells secrete angiogenic cytokines, such as vascular endothelial growth factor (VEGF), platelet-derived growth factor (PDGF) and basic fibroblast growth factor (bFGF) that stimulate neovascular formation and inflammatory cytokine, such as TNF-α and IL-1β. We measured these serum levels in patients with glial cell tumors and meningioma.

**Materials and Methods:**

This was a case-control study in 2014-2015 on patients diagnosed with meningioma/glioma. All demographic and clinical data were registered. The tumor volume and intraoperative bleeding were recorded. Serum levels of VEGF, PDGF, FGF, TNF-α and IL-1β were measured by ELISA methods.

**Results:**

Ninety-six patients were enrolled in this study, 32 in each group. Patients VEGF level with cranial tumor, glioma/meningioma had increased. VEGF level was highest among grade IV tumors, larger tumors, and in glioblastoma multiform. There was an upsurge in VEGF serum level as glioma grade increased. The highest VEGF levels were seen in parasagittal meningioma. In contrast to VEGF, PDGF was slightly elevated in glial cell tumors, which was significantly elevated in meningioma. Higher PDGF correlated with increased intraoperative bleeding, especially in meningioma cases. Oligodendroglial tumors expressed higher PDGF levels in contrast to other glial tumors. FGF level was not statistically significant. TNF-α and IL-1β expressions were significantly higher in the meningioma and glioma group in comparison to control group.

**Conclusion:**

We found increased VEGF and PDGF serum levels in CNS patient’s tumor. A different role for PDGF was found in the pathogenesis of neovascularization of meningioma, as well as oligodendroglioma. No significant result was found for FGF. TNF-α and IL-1β can serve as key prognostic biomarker in high-grade glioma and meningioma patients.

## Introduction

Cancer is a prevalent health concern around the worldwide, and no race, nationality, or social class is free from it. It has various types with varying incidence rate in different populations, and some types are rare in different parts of the world. In developing countries, cancer is still a significant health problem, and is likely to increase in the future.

Meningioma and glioma are the most common primary central nervous system tumors in adults. Most meningioma do not reoccur, if complete surgical dissection is performed, but different subset of recurrent aggressive meningioma (Grade II and III) presents with high mortality (Chen et al., 2013; Peyre et al., 2015). In contrast, glioma patients have a survival rate of approximately 2 years after diagnosis (Chen et al., 2013). 

Studies on cancer in developing countries, such as Iran are limited due to lack of instruments, disease control and surveillance. Among the top 10 cancers for both males and females in Fars province, southern Iran, nervous system ranks fifth. The crude incidence rate and age specific rate (ASR) of nervous system cancers amongst male population in Fars Province was reported 1.73 and 2.08, while these figures among females were 1.23 and 1.46 (Mehrabani et al., 2008).

The feature of brain cancer is the presence of high vascularity (Hoelzinger et al., 2007). Tumor growth and expansion depend on the tumor ability to generate its own supportive vascularization. Every tumoral process is in a serious need of blood vessels formation, while in the absence of vascularization, no tumor can grow larger than 1-2 millimeters. This issue is of a great importance, especially in central nervous system tumors. Glioblastoma is an example of a highly vascular tumor with an extensive angiogenic component driven by VEGF-A (Holash et al., 1999). It was shown that VEGF inhibition might promote tumor cell invasiveness (Du et al., 2008). 

Several factors affect new blood vessel formation including vascular endothelial growth factor (VEGF) platelet-derived growth factor (PDGF), and basic fibroblast growth factor (bFGF), while many factors were reported to inhibit neo-vascularization, such as endostatin, thrombospondin, and soluble VEGF-receptor. These factors secrete in specific circumstances; for instance, there is a rise in VEGF secretion with hypoxia in tumor cells.

Hypoxic tumor cells secrete angiogenic cytokines, such as VEGF, PDGF, bFGF and insulin growth factor (IGF) to stimulate neovascular formation(Carmeliet and Jain, 2000). PDGF is a disulfide-linked dimer and polypeptide chains synthesized by platelets, macrophages, epithelial and endothelial cells that in addition to its direct effect on tumor growth, PDGF has also an angiogenic activity (Li et al., 2000). Autocrine and paracrine loops of PDGF secretion were described in glioma and malignant astrocytomas (Lokker et al., 2002). In the case of meningioma, it was found that growth stimulation was completely stopped by an anti PDGF antibody (Li et al., 2007). Todo et al., (1993) showed that PDGF antagonist could block DNA synthesis in meningioma tumor cell lines. Therefore, PDGF inhibition might be an attractive therapy option, alone or combined with surgery or/and radiotherapy in refractory cases. It was shown that combined VEGF and PDGF inhibition signaling caused tumor vessel regression by direct anti-angiogenic effect to both endothelial cells and pericytes (Bergers et al., 2003). VEGF inhibition in endothelial cells decreased the endothelial cell survival, proliferation, canalization and invasion in vitro (Bergers et al., 2003). Among several inflammatory cytokines shown to be over expressed at tumoral level, TNF-α and IL-1β are closely related to pain (Albulescu et al., 2013).

Numerous studies have shown that interleukin-1 beta (IL-1b), a pro-inflammatory cytokine in chronic inflammation has a higher level in several tumor site, including meningioma, which plays a critical role in tumor growth, metastasis, and angiogenesis (Apte et al., 2006; Multhoff et al., 2012). 

The present study was performed to determine the expression of VEGF, PDGF, FGF and inflammatory cytokines in patients with glial cell tumors and meningioma. 

## Materials and Methods

*Patients and sample collection*

This case-control study was conducted on a population of patients who referred to Chamran Hospital, Shiraz, southern Iran during 2014-2015, with the diagnosis of brain tumor, either meningioma or any other type of glioma. Total sample size was 96 patients, 32 in each group. This study was approved by the local ethics committee of Shiraz University of Medical Sciences, Shiraz, Iran. Written informed consent was obtained from each participants.

Diagnosis was made by brain CT-scan and MRI, and confirmed post-operatively by pathology. Brain tumor patients were admitted pre-operatively, and underwent a thorough neurological examination to find any evidence of increased intracranial pressure (ICP), such as papilledema. All other demographic and clinical data including age, gender, date of admission and operation, symptoms, family, and other pertinent histories were also obtained and recorded. Tumor volume was determined based on volumetric studies on the brain CT and MRI finding. 

All patients underwent neurosurgical operation. A sample from their tumoral tissue was sent for pathological examination. The amount of intraoperative blood loss was also recorded as a crude index of tumor invasiveness, and vascular proliferation. A control group consisting of 32 road accident patients with no history and sign and symptoms of autoimmune diseases, rheumatology diseases or malignancies were included. 

*Angiogenic and Inflammatory cytokines analysis*

After confirming patients’ tumor, a blood sample was drawn from each participant to measure the serum levels of vascular endothelial growth factor (VEGF), platelet-derived growth factor (PDGF), and fibroblast growth factor (FGF) using ELISA kit (VEGF, PDGF, FGF; R and D, Minneapolis, Minnesota, United States), TNF-α and IL-1β were using ELISA kits (eBioscience, USA) according to the instructions provided by the manufacturers.

**Figure 1:**
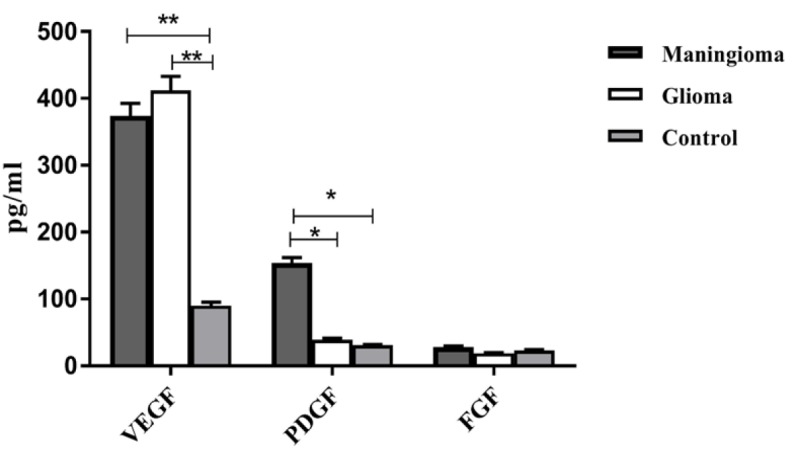
The Angiogenic Cytokine Levels of VEGF, PDGF and FGF in Different Study Groups. The comparison of the levels of VEGF, PDGF and FGF in the patients with meningioma, glioma and the control group

**Figure 2:**
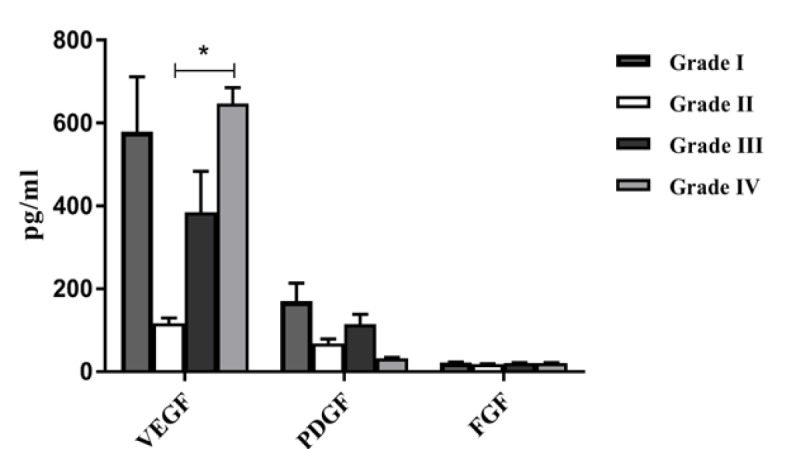
The Angiogenic Cytokine Levels of VEGF, PDGF and FGF in Different Pathological Grades. The comparison of the levels of VEGF, PDGF and FGF in brain tumor patients with different pathological grades

**Figure 3:**
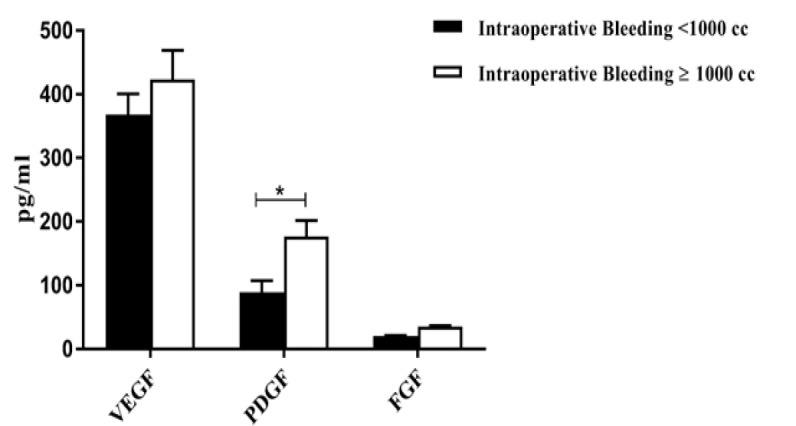
The Angiogenic Cytokine Levels of VEGF, PDGF and FGF in Tumors with Intraoperative Bleeding. The comparison of the levels of VEGF, PDGF and FGF in tumors with intraoperative bleeding of more or less of 1000 ml

**Figure 4:**
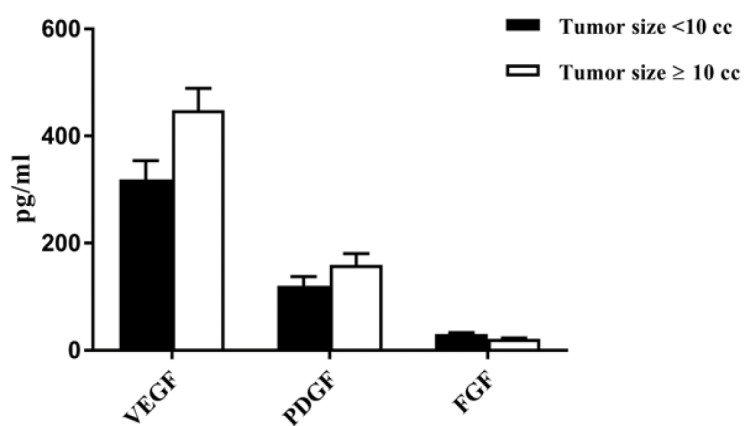
The Angiogenic Cytokine Levels of VEGF, PDGF and FGF in Different Tumors Size. The comparison of the levels of VEGF, PDGF and FGF in tumors smaller or larger than 10 ml

**Figure 5:**
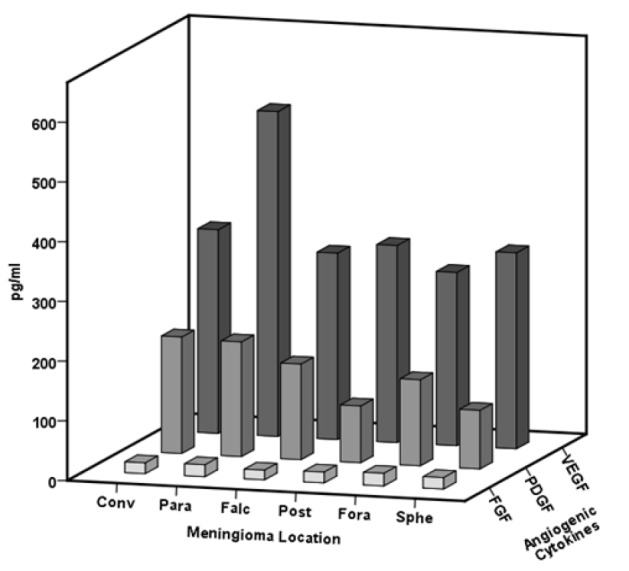
The Angiogenic Cytokine Levels of VEGF, PDGF and FGF in Different Meningioma Location. The comparison of the levels of VEGF, PDGF and FGF in different meningioma locations. VEGF level significant increase in parasagittal meningiomas. Meningioma location: convex (Conv), parasagittal (Para), falcine (Falc), posterior fossa (Post), foramen magnum (Fora), sphenoid wing type (Sphe)

**Figure 6:**
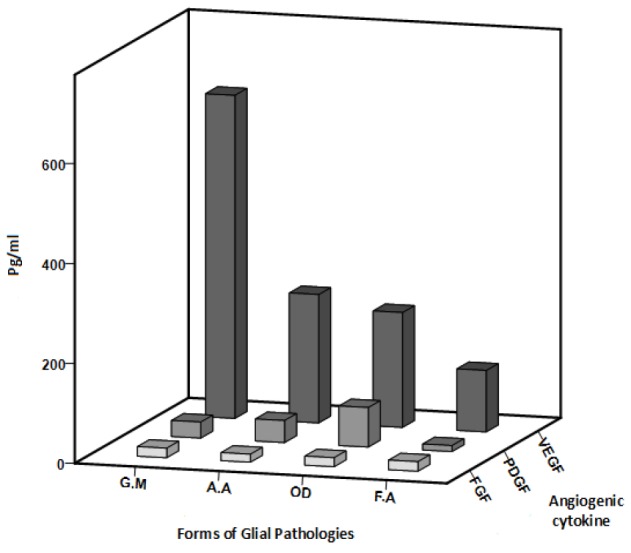
The Angiogenic Cytokine Levels of VEGF, PDGF and FGF in Different forms of Glial Pathologies. The comparison of the levels of VEGF, PDGF and FGF in different forms of glial pathologies. VEGF level significant increase in glioblastoma multiforme and PDGF level significant increase in oligodendroglioma. forms of glial pathologies: glioblastoma multiform (GM), anaplastic astrocytoma (AA), oligodendroglia (OD), fibrillary astrocytoma (FA)

**Figure 7:**
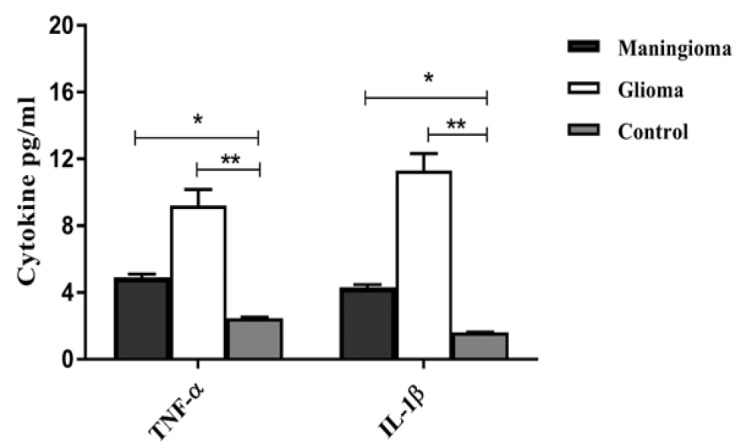
The Inflammatory Cytokine Levels of TNF-α and IL-1β in Different Study Groups. The comparison of the levels of TNF-α and IL-1β in the patients with meningioma, glioma and the control group

**Figure 8:**
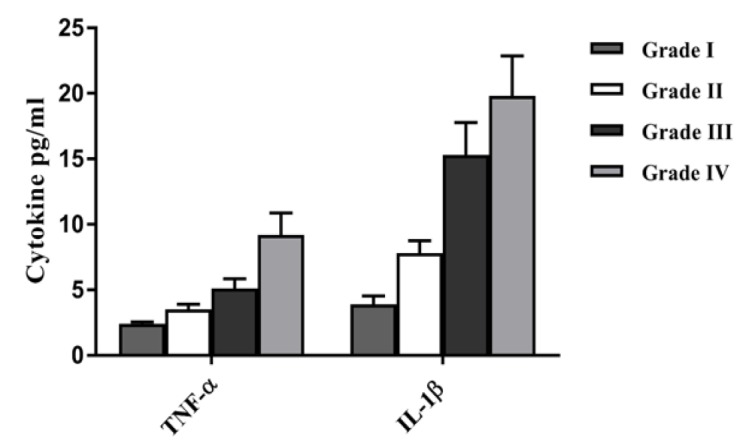
The Inflammatory Cytokine Levels of TNF-α and IL-1β in Different Pathological Grades. The comparison of the levels of TNF-α and IL-1β in brain tumor patients with different pathological grades

**Table 1:** Comparison of Different Parameters, Gender/ Age Related to the Patients with Brain Tumor and the Control Groups

Characteristics	Glioma	Meningioma	control	P value
Sex (Male/ Female)	1.7	0.43	2.7	0.01
Age (mean ± SD)	27.3 ± 7.2	48.7 ± 8.9	34.4 ±10.1	0.03
Mean blood Loss (Min-Max) (cc)	940 (400-2,300)	1,280 (50-5,000)	-	0.04
Blood loss <1,000	73.30%	52.20%	-	
Blood loss ≥1,000	26.70%	47.80%	-	
Tumor size <10	50%	60%	-	0.1
Tumor size ≥10	50%	40%	-	

*All collected data were analyzed using SPSS software version 23.0 (Washington, USA) for Windows*

The Chi-square test was used to evaluate the qualitative data (age, gender, etc.). The Kruskal–Wallis test was used for the statistical analysis of the data for all groups. The Mann–Whitney test with the Bonferroni correction was used for the comparison between each group.

## Results

Ninety-six patients were enrolled in this study, 32 participants in each group, the mean age was 36.8±10.9 years. Male patients were 48.3% and female 51.7% of the population. There was a significant difference in gender, mean age, and blood loss range between patients and the control group (Table 1).

The mean tumoral volume was 9.4 ml measured by volumetric technique on imaging studies, which was determined intraoperatively. Glial tumors were generally larger than meningioma; however, such trend was not statistically significant (p=0.1).

Meningioma was significantly associated with bleeding in comparison with glial tumors (1280 vs. 940 ml; p=0.04). Also, a large number of meningeal tumors (nearly half) had more than 1,000 ml bleeding, which was considered as a critical point during operation (Table 1). Papilledema was observed in 31.2% and 21.9% of glioma and meningioma patients, respectively, but there was no significant trend for papilledema to occur in any specific group (p=0.3).

Patients were divided according to types of meningioma based on location, and the convex meningioma was the most common type (31.2%), followed by parasagittal (18.8%), sphenoid wing type (15.6%), falcine (12.5%), posterior fossa (12.5%), foramen magnum (6.3%), and petroclival (3.1%), according to WHO classification (Louis, 2007). Grade one consisted of the most common (21-65.6%) tumor grade. Similarly, patients with glioma tumor were classified according to the constituent cells of the central nervous system, and glioblastoma multiform was the most common type (46.8%), followed by oligodendroglia (21.9%), fibrillary astrocytoma (18.8%), and anaplastic astrocytoma (12.5%). According to WHO grading system, grade 4 was the most common in glioblastoma multiform, grade three in anaplastic astrocytoma, two in fibrillary astrocytoma, two and three in oligodendroglia.

As Figure 1 shows, significantly higher VEGF level in the glioma and meningioma compared to the control group (P=0.008, p=0.005, respectively) was observed. In the meningioma group, PDGF level was statistically higher than glioma and the control group (p=0.01, p=0.02, respectively). There was no statistically significant difference in FGF level among glioma and meningioma, and the control group (P=0.3).

The mean VEGF serum levels in different pathological grades is shown in Figure 2. VEGF was significantly higher in grade IV tumoral lesions in comparison with grade II (p=0.03). PDGF and FGF levels were not significantly different in any of the grades (P=0.4, p=0.6, respectively).

As seen Figure 3, the mean serum PDGF level in tumors with intraoperative bleeding of more than 1,000 ml, was significantly higher than other groups (P=0.04). However, VEGF and FGF levels represented no significant difference between the two groups (p=0.6, p=0.7, respectively). 

The mean serum VEGF, PDGF and FGF levels were compared in tumors smaller or larger than 10 ml. Even though no significant difference was found between the two groups; however, VEGF levels were higher in larger size tumors, and the difference was not statistically significant (p=0.3). PDGF and FGF were not statically different (p=0.3, p=0.5, respectively) (Figure 4).

Mean tumor volume was 9.4 cc as measured by volumetric techniques on imaging studies, which was also roughly determined intraoperatively. In general, Glial tumors were larger than meningioma, but such trend was not significant (p=0.1) (Table1).

Meanwhile, VEGF and PDGF had increased in different meningioma locations, but significant higher VEGF level was observed in parasagittal meningioma (p=0.04). FGF was not statically different among groups (p=0. 8) (Figure 5). 

In all forms of Glial pathologies, VEGF serum level had increased, but the observed increase was only significant in Glioblastoma multiform (p= 0.02). However, in oligodendroglioma PDGF level increased significantly (p= 0.03). FGF level was not statistically significant in none of the groups (p=0.7) (Figure 6).

We observed significantly higher TNF-α level in the glioma and meningioma group in comparison to the control (P=0.009, p=0.04). In the meningioma and glioma group, IL-1β level was statistically higher than the control group (p=0.03, p=0.001) (Figure 7). 

The mean TNF-α and IL-1β serum levels in different pathological grades are shown in Figure 8. TNF-α and IL-1β were higher in high grade tumoral lesions in comparison with low grade ones. TNF-α levels had increased significantly in Glioblastoma multiform (p= 0.03).

## Discussion

Recent funding in research related to the discovery of specific tumor biomarkers, which are significant for effective diagnosis and prognosis are beginning to payoff. Key biomarkers can theoretically overshadow traditional radiological or pathological methods by permitting specificity of early detection, when joined with tumor molecular profiling and clinical associations.

This case-control study was conducted to evaluate the mean serum level of different angiogenic factors as well as the relationship between groups and multiple tumor characteristics; including size, pathology, grade, and the amount of intraoperative bleeding. 

The result showed increased VEGF level in patients with cranial tumor, either glioma or meningioma, which was in line with previous studies. Hence, VEGF expression was higher in high grade meningioma in comparison with low-grade meningioma (Dharmalingam et al., 2013; Reszec et al., 2015), as well as in microvascular density, microvascular morphology in glioblastoma (Barajas et al., 2015). 

Remarkably, VEGF level was highest among grade IV tumors, namely glioblastoma multiform as one of the most important vascular tumors, while vascular proliferation was considered as an important marker for its pathologic diagnosis. Such association confirms the role of VEGF in forming new fragile vessels. Similarly, Dharmalingam P et al., showed that total of Grade II and Grade III meningioma were VEGF positive, while only 65% of Grade I tumors were positive (Dharmalingam et al., 2013).

VEGF was higher in larger tumors, which might be an indication that the tumor itself produces VEGF. But the relationship between VEGF and tumor size was not significant, which might be due to the absence of a logic or statistical cutoff point for tumor size (O’Connor and Jayson, 2012).

VEGF level did not differ significantly in patients with small or large amounts of intraoperative bleeding. This might be due the absence of a logical or statistical cutoff point for the amount of bleeding, and our result was consistent with previous studies (Karsy et al., 2017). In addition, Sonoda et al., (2003) observed a correlation between higher serum VEGF with grade tumor. 

Another interesting finding was the increasing trend of serum VEGF levels as glioma grade increased. This was in line with the increase in vascularity of the tumor as the grade augmented (Flynn et al., 2008). As stated earlier, the highest impact was evident in GBM group. Sonoda et al., (2003), suggested that VEGF121 and VEGF165 isoforms might contribute to glioma vascularization, oxygenation, and growth.

In meningioma group, the most significant result was observed with parasagittal meningioma; however, the reason was not evident, and the proximity of this type of meningioma to the venous sinuses might underlie such relationship. In contrast to VEGF that was higher than the control in both groups of brain tumors, PDGF was slightly elevated in glial cell tumors, and it was significantly elevated in patients with meningioma. Our finding was in agreement with previous observations stating that PDGF ligand and receptor were both expressed in surgical specimens of meningioma (Andrae et al., 2012). This autocrine loop for PDGF secretion was also confirmed by our results, which is unique to meningioma.

Data points to another pathological difference between meningioma and gliomas. The vessels in meningioma are not fragile as is the case with glioma, and are hyalinized thick vessels contributing to a well-organized vascular network in meningioma, which leads to the classic star-burst view in angiography. Such a difference in vasculature can be explained by the dominance and specificity of PDGF in meningioma (Jain, 2005). Heightened platelet-derived growth factor (PDGF) signaling are normally obserproneural subtype of glioblastoma and can drive gliomagenesis (Rahme et al., 2016), which was proven in our study. Another explanation for higher PDGF levels in meningioma might be its mesenchymal origin. PDGF is also secreted from stem cells of mesenchymal origin (Crisan et al., 2008), and this can prove the contribution of stem cells to the formation and growth of meningioma.

No correlation was observed with pathological grades, which might be due pathological grades in our study that were mainly distributed in glioma patients. Meningioma in our group was mainly of type one, and did not have any significant effect on the data. Similar to VEGF, larger tumors were linked to higher PDGF levels, which was not significant. Peyre et al., (2015) showed that the crucial role of PDGF-B in meningioma is the initiation and progression. Our finding showed that higher serum PDGF levels were correlated with higher amount of intraoperative bleeding, especially in meningioma. Many reports have shown that higher PDGF levels led to a more organized vascular network in meningioma (Jiang et al., 2002; Jain, 2005). Although the vessels were less fragile than glioma vessels, intraoperative bleeding was significantly higher in meningioma, which can be explained by higher PDGF levels in these cases. Another significant result was observed in oligodendroglial tumors expressing higher PDGF levels as an exception in glial tumor patients (Ohgaki and Kleihues, 2005). This finding might be due to a mesenchymal stem cell origin of these tumors. As stated earlier, PDGF is actively secreted by stem cells from mesenchymal (Ponte et al., 2007). 

Our study did not find any significant difference between FGF level in the meningioma and glioma in comparison with the control group. Similarly, Ribom et al., (2003) also measured FGF-2, but at an extremely low concentrations in only 2 out of 7 patients. Consistent with the current study, Sun et al., (2010) showed significantly increased PDGFR-α and FGF-2 levels in recurrent tumors, and the important role of PDGFR-α and FGF-2 in the angiogenesis, and recurrent craniopharyngiomas. These growth factors are not released into the CSF in any amount (Beenken and Mohammadi, 2009), and might not be suitable as a biological markers.

Other findings, were increased levels of IL-1β in the sera of glioma and meningioma patients that strongly correlated with tumor grade and clinical aggressiveness in glioblastoma.

Previous studies showed that increased secretion of IL-1β by glioma cells and in patients with IL-1β positive tumors had generally worsen the prognoses (Lewis et al., 2006; Yeung et al., 2012). These cytokines are related to the development of glioblastoma multiform (Albulescu et al., 2013), which is in line with our finding.

Elevated TNF-α concentrations were observed in meningioma and glioma patients. TNF-α induces IL-6 synthesis and stimulated antigenic factors, such as VEGF. Also this cytokine is usually involved in tumor cell invasion and metastasis. This finding was also observed in our study. 

Finally, we found increased VEGF and PDGF serum level in CNS patients’ tumor. A different role for PDGF was found in the pathogenesis of neovascularization for meningioma as well as oligodendroglioma. No significant result was found for FGF. TNF-α and IL-1β can serve as key prognostic biomarker in high-grade glioma and meningioma patients. Our findings propose a direct connection between the pathologic behavior of tumor and vascularization. In addition, further studies can create a multimolecular panel for a more suitable patient stratification and more acceptable therapeutical methods.

The contribution of cytokines in inflammation and pain, as trustworthy targets for examination, with possible diagnostics and therapeutic applications.
